# Persistence of Contrasting Traditions in Cultural Evolution: Unpredictable Payoffs Generate Slower Rates of Cultural Change

**DOI:** 10.1371/journal.pone.0099708

**Published:** 2014-06-18

**Authors:** Christine A. Caldwell, Roland M. Eve

**Affiliations:** Psychology, School of Natural Sciences, University of Stirling, Stirling, United Kingdom; Durham University, United Kingdom

## Abstract

We report an experimental test of the hypothesis that contrasting traditions will persist for longer, maintaining cultural differences between otherwise similar groups, under conditions of uncertainty about payoffs from individual learning. We studied the persistence of two alternative, experimentally-introduced, task solutions in chains of human participants. In some chains, participants were led to believe that final payoffs would be difficult to predict for an innovative solution, and in others, participants were aware that their final payoff would be directly linked to their immediate solution. Although the difference between the conditions was illusory (only participants’ impressions were manipulated, not actual payoffs) clear differences were found between the conditions. Consistent with predictions, in the chains that were less certain about final payoffs, the distinctive variants endured over several replacement “generations” of participants. In contrast, in the other chains, the influence of the experimentally-introduced solutions was rapidly diluted by participants’ exploration of alternative approaches. The finding provides support for the notion that rates of cultural change are likely to be slower for behaviors for which the relationship between performance and payoff may be hard to predict.

## Introduction

Theoretical models predict that individuals should (adaptively) increase their reliance on social learning where the potential payoffs from individual learning are uncertain, or difficult to predict [1], [2], and that the outcome of such a trade-off can be, under some circumstances, suboptimal traditions [3], [4]. The possibility of suboptimal outcomes arises because an increased reliance on social learning within a population entails a reduction in innovation, and therefore less exploration of potentially superior alternatives. Of course, the behavior of the population is generally expected to move in the direction of optimality, but an increased reliance on social learning would be expected to result in slower rates of cultural change. Since the degree of reliance on social learning may be a function of the predictability of the payoff, it follows that payoff predictability may be an important determining factor with regard to the longevity of particular behavioral traditions. In the current study, we have taken an experimental approach to the question of strategic trade-offs between social and individual learning, and the consequence of this balance for the persistence of particular cultural traits. Our expectation is that rates of cultural change will be slower when payoffs are more difficult to predict.

Experimental approaches to studying human culture have become increasingly common in the recent evolutionary literature [5], [6], [7]. These methods involve chains of participants taking part in the experimental task in succession, with opportunities to interact with or observe their immediate predecessors. However, to date there are few such studies focused on the question of the persistence or otherwise of group-specific traditions.

In an early example of this approach, Jacobs and Campbell [8] investigated the persistence of a counterintuitive belief (introduced by experimental confederates) within chains of participants. Jacobs and Campbell concluded that all persistent cultural beliefs (even demonstrably counterfactual ones, such as superstitions) must have some inherent value, as social influence alone was insufficient to support arbitrary traditions in the face of “continuous spontaneous innovation in a natural direction” (p657). Following this up, Weick and Gilfillan [9] found that an experimentally introduced (effective) strategy for solving their experimental task was faithfully transmitted over multiple generations, whereas a more complicated alternative was rapidly abandoned.

In previous studies chains of participants (or laboratory “microsocieties”, [5], [8]) have been presented with simple building tasks with clear objective goals (building paper airplanes to fly as far as possible, and building towers from raw spaghetti to be as tall as possible) [10], [11], [12]. In these experiments, participants were not exposed to any experimental manipulation regarding the initial solution. The first participant therefore cannot use social information at all, but later participants can make use of information gleaned from observing earlier efforts from their own chain. In these studies it was found that social and individual learning were combined adaptively by the participants, to produce the “ratcheting” characteristic of human cumulative culture [13], with solutions becoming increasingly effective over generations. This adaptive integration of social learning and innovation was accompanied by some evidence of contrasting design traditions. Caldwell and Millen [10] found that participants’ tower and plane designs were more similar to other designs from their own chain, compared with those from different chains.

Furthermore, in a condition in which personal payoffs were more difficult to predict (because tower height was measured after a delay and some perturbation of the tower), traditions appeared stronger, implying a greater reliance on social learning over innovation. However, in this previous study, the persistence of traditions could only be inferred from the measures of within-chain and between-chain similarity. It was not possible to track the longevity of particular design features as there was no experimental manipulation of the tower designs to which participants were exposed. As a result of this, similarity was measured by using the subjective ratings of naïve coders asked to compare pairs of towers [12]. It therefore remains possible that the higher similarity ratings in the unpredictable payoffs condition were not a result of higher fidelity copying, but an outcome of certain designs being preferred, or a smaller range of design types being preferred, in the unpredictable payoffs condition, in which the importance of tower stability was strongly emphasized [12].

Our aim in the current study was therefore to implement a direct experimental test of the hypothesis that particular designs will persist for longer when the likely payoffs for innovative strategies are difficult to predict, using the spaghetti tower task from previous experiments [10], [12]. To do this we have experimentally manipulated the initial solutions presented to the early generation participants of each chain, exploiting the logic of “two-action” designs, such that it is possible to track the influence of our two alternative “seed” solutions along the chains. A previous study using the same two alternative designs in the context of a dyadic social learning experiment [14] established that one of these alternatives was objectively suboptimal to the other. This therefore offered the additional possibility of tracking the relative rates of change of the two tower types (of varying effectiveness) under different payoff conditions. In the current study we also wanted to ensure that participants’ primary focus was their performance on the task, rather than, for example, fulfilling the expectations of fellow group members. Payment for participation was therefore directly (and steeply) related to task score, and participants did not take part in the task face-to-face, but were simply shown photographs of their predecessors’ solutions. In addition, we created the two payoff conditions such that there was no difference between them other than participants’ impressions about the directness of the relationship between immediate solution and final payoff. The apparent difference between the conditions was therefore entirely illusory.

We predicted that participants would show significant matching to the seed variant for their chain, compared with the alternative seed. We also predicted that copying would be stronger in the condition in which the relationship between solution and payoff was more unpredictable, and that this would result in greater persistence of the features of seed designs in later generations of chains in this condition. Finally, we predicted any differences in persistence of the designs between the predictable and unpredictable payoff conditions would be most marked for the suboptimal tower design, which was expected to be more rapidly modified in the predictable payoff chains.

## Materials and Methods

### Ethics Statement

Ethical approval for this research was provided by the University of Stirling Psychology Ethics Committee. The procedure was explained to all participants in advance, and each gave written consent to participate. All participants were over 16 years of age and were therefore able to give full informed consent.

### Design

**Figure 1:**
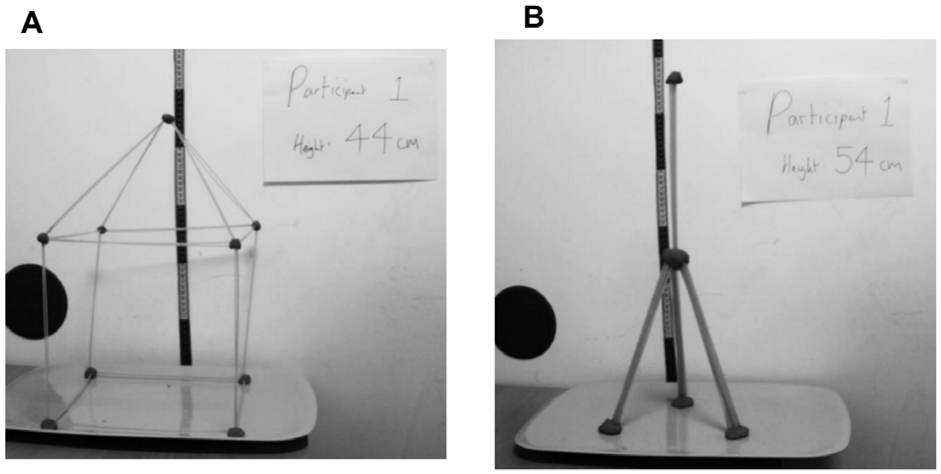
Examples of the seed towers: cubic (A) and tripod (B) designs.

Participants were assigned to one of twenty chains of participants, with five participants to each chain (see Table 1). The chains were seeded with towers which had in fact been built by the experimenter to a specific design. Half of the chains were seeded with photographs of one type, and the other half were seeded with photographs of a contrasting design. Examples of seed photographs of each design (which we have labeled “cubic” and “tripod”) are shown in Fig. 1. These two alternative tower designs were selected because they represented fairly typical tower types based on previous experiments using this task which relied solely on participants’ spontaneous design choices, without interference from designs created by an experimenter [10], [12]. These two designs are also distinctly different, with several features that can be identified to distinguish them. This permitted an objective coding scheme to be developed, based on these contrasting features (Table 2).

**Table 1:** The chain design.

Participant Number	Solutions Viewed
P1	Seed 1, Seed 2
P2	Seed 2, P1
P3	P1, P2
P4	P2, P3
P5	P3, P4

**Table 2:** The coding scheme used to quantify the similarity of participants’ towers to the two seed tower types.

Tower Feature	Cubic	Tripod
**Base contact**	Modeling clay and spaghetti (1)	Modeling clay only (1)
**Base shape**	Square (1)	Triangular (1)
**Lower level structure**	Vertical from modeling clay contactpoints, not converging to singlepoint (1)	Approximately vertical converging to single point (1)
**Upper level structures**	Horizontal joins between verticaluprights (0.5), and converges to asingle point at highest point (0.5)	Single vertical element as highest level (0.5), with any/all upper levels as single verticals (0.5)

Numbers indicate the points attributed to towers displaying those features as their cubic and tripod feature scores.

Seed tower type, and payoff predictability were manipulated independently of each other, so five replicate chains were run in each of the four resulting conditions: cubic unpredictable; cubic predictable; tripod unpredictable; tripod predictable.

### Participants

One hundred participants were recruited to take part in the study, 66 of whom were recruited on campus at the University of Stirling, and 34 of whom were recruited at Glasgow Science Centre (http://www.gsc.org.uk). Initially we ran twenty chains of five participants, with five chains in each of the four conditions. Within the predictable payoffs condition, there was an even balance of University to Science Centre participants (25∶25). However, in the unpredictable condition, only nine participants were run at Glasgow Science Centre. These were confined to two chains (one cubic and one tripod). In the Science Centre environment it proved rather more difficult to generate the intended level of apprehension about the purported structural tests (see Procedure section for details of the unpredictable condition manipulation). Testing was carried out behind a makeshift visual barrier, rather than in a purpose-built participant testing room, as was used for University participants, and an apparent lack of any additional equipment may have seriously affected the believability of this manipulation. Subsequent analyses corroborated these impressions, indicating that these participants performed differently to those recruited on campus in the same condition. These two chains were therefore excluded from the analyses reported here. However, it should be noted that all analyses have also been performed without exclusion, with no change to the significance or otherwise of the results. Of the 90 non-excluded participants, 60 were female and 30 male. Their mean age was 23.5 years (*SD* = 6.3).

All participants took part in return for an incentive fee, which was determined by their success on the task. On top of a base rate of £1, they were paid 50 pence for every 10 cm of height achieved. The mean sum earned was £3.14, the maximum was £7, and the minimum was the base fee of £1. Participants recruited at the University were paid in cash, and those recruited at Glasgow Science Centre were paid in vouchers which could be exchanged for food and drink at the on-site café. Science Centre vouchers were provided in increments of one pound, so participants whose earnings were not an exact multiple of one pound had their fee rounded up to the nearest whole pound (but were unaware that this would be the case until after the experiment had been completed).

### Apparatus

Each participant was provided with one 500 g packet of spaghetti, plus approximately 200 g of red Newplast modeling material.

### Procedure

Participants were shown photographs of the towers built by the previous two participants in their chain. The first participant in each chain was shown the two seed towers (see Design section). The second participant in each chain was shown only one of the seed photographs, plus the photograph of the tower built by the first participant. The third, fourth and fifth participant in each chain were shown only photographs of towers built by real participants (see Table 1).

Participants were informed that the goal of the spaghetti tower building task was to build a tower as tall as possible using the spaghetti and modeling clay. They were also informed that they would be shown photographs of the solutions of two previous participants, and that they would be given three minutes to study them and develop a strategy for their own tower, prior to a seven minute building period in which they would complete their own tower. In the predictable payoffs condition, participants were simply told that the reward system was related to the height of their tower, and that they would receive 50 p for every 10 cm of height achieved, on top of their £1 base fee. In the unpredictable payoffs condition, participants were given identical information about the reward structure, but were also told that upon completion of their tower they would be asked to leave the testing area while their tower underwent a series of structural tests which would last around five minutes. It was emphasized that only after these tests had been completed would the tower be measured and the final payment determined, and that should their tower fail the tests and collapse, this would result in a corresponding reduction in the final payment. It was also emphasized to participants in the unpredictable payoffs condition that photographs were taken after the structural tests had been completed, so the photographs that they were shown depicted the final height of those towers. In reality no such structural tests were completed, as the experimenter simply sat in the testing area with the completed tower for the specified period without touching it, so in effect rewards were determined in an identical manner in both conditions.

Photographs were taken with a measuring tape in the background, and with the height measurement clearly displayed alongside the tower (Fig. 1).

### Data Coding and Analysis

Towers built by participants were coded according to the features they had in common with the two alternative seed towers. Features were coded as either cubic-like, or tripod-like (Table 2). All photographs were coded according to this scheme by both authors working independently. The ratings showed high concordance, suggesting that the scores could be assigned with high reliability using this coding scheme (Spearman’s Rho for cubic features: *r* = .909, *n* = 100, *p*<0.0005; for tripod features: *r* = .942, *n* = 100, *p*<0.0005).

In the predictable payoff condition, participants from the two different recruitment sites (see Participants section) exhibited comparable levels of copying, and built towers of equivalent height, so these data were combined in subsequent analyses. Where data were non-normally distributed (exhibiting significant skewness and/or kurtosis) nonparametric statistics were used. Two-tailed probabilities are reported.

## Results

### Individual-level Copying of Observed Solutions

The degree of overlap between any given tower and its two immediate predecessors (i.e. those the participant had the opportunity to view) was calculated in order to give a measure of direct copying from observed solutions. We used the proportion of features shared between any two towers, in relation to the total number of features (shared + unshared) exhibited across both towers, as well as the absolute number of shared features. Participants in the unpredictable condition matched the towers they were shown significantly more than those in the predictable condition. For the absolute number of shared features, in the unpredictable condition the mean was 2.41 (*SD* = 1.11), compared with 1.77 (*SD* = 1.09) in the predictable condition (*t* = 2.753, *df* = 88, *p* = 0.006). For the proportional overlap measure, the mean was 0.53 (*SD* = 0.28) in the unpredictable condition and 0.39 (*SD* = 0.25) in the predictable condition (*t* = 2.364, *df* = 88, *p* = 0.020).

### Matching to Seeds

The degree of matching to original seed towers was analyzed over chains using repeated measures analyses. The average number of features matching the original seed tower was calculated for each chain, for its early generation (1 and 2, exposed to the seeds) and late generation (3, 4 and 5, not directly exposed to the seeds) participants. The corresponding descriptive statistics are displayed in Table 3. A 2×2×2 ANOVA was performed, with generation (early; late) as a repeated measures variable, and payoff condition (predictable; unpredictable) and seed tower type (cubic; tripod) as between-subjects variables. There was a main effect of generation, with early generation towers matching more closely to their original seed than late generation towers (*F*_1,14_ = 9.924, *p = *0.007). In line with the hypothesis there was also a main effect of payoff condition, with stronger matching to the seed in the unpredictable condition (*F*_1,14_ = 8.744, *p = *0.010). There was no main effect of tower type (*F*_1,14_ = 0.810, *p = *0.383), indicating that the two designs were equally well copied. Interactions were all non-significant. For generation by payoff condition, *F*_1,14_ = 0.169, *p* = 0.687; for generation by tower type, *F*_1,14_ = 0.300, *p* = 0.592; for payoff condition by tower type, *F*_1,14_ = 1.545, *p* = 0.234; and for the three-way interaction between generation, payoff condition, and tower type, *F*_1,14_ = 0.300, *p* = 0.592.

**Table 3:** Mean features matching original seed tower, and mean tower heights.

Payoff Condition	TowerType	Features Matching OriginalSeed Tower (see Table 2)		Height (cm)	
		Early Gens.	Late Gens.	Early Gens.	Late Gens.
**Unpredictable**	**Cubic**	3.00 (0.46)	2.50 (0.36)	32.75 (14.71)	36.50 (9.29)
	**Tripod**	2.38 (0.92)	1.88 (0.16)	60.00 (10.90)	48.58 (10.81)
**Predictable**	**Cubic**	1.95 (0.94)	1.10 (0.84)	41.80 (15.11)	43.13 (17.48)
	**Tripod**	1.85 (0.95)	1.40 (0.53)	53.80 (14.62)	56.73 (7.70)

Early indicates generations 1 & 2, and Late indicates generations 3, 4 & 5. Predictable and Unpredictable indicate the two different payoff conditions, and Cubic and Tripod indicate the two different seed tower conditions. Standard deviations are given in parentheses.

**Figure 2:**
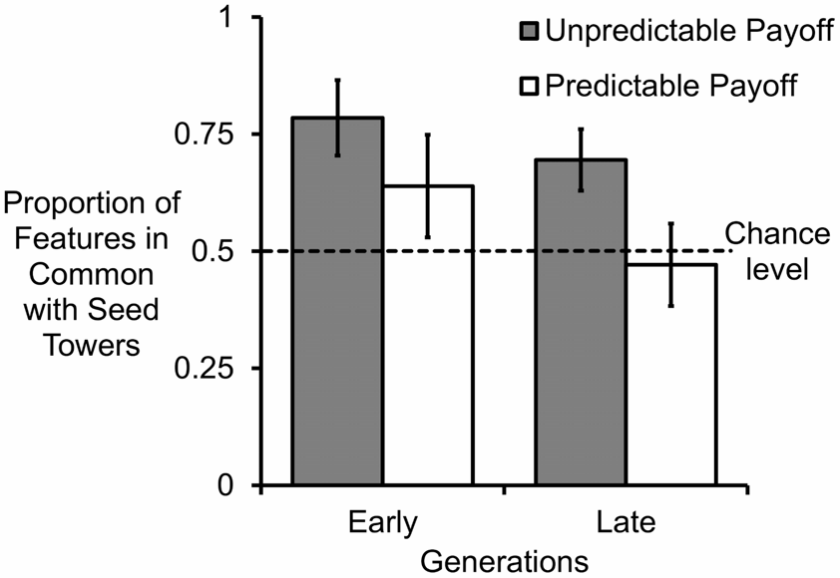
Mean match proportion scores (+/−1*SE*) for the two payoff conditions. Early indicates generations 1 & 2, and late indicates generations 3, 4 & 5.

In addition, in order to test for above chance-level matching to seed type, all towers were given a score to indicate the proportion of features they had in common with their seed tower, in relation to the total number of features that they had in common with either seed type. Scores were therefore calculated as: features shared with seed tower/(features shared with seed tower + features shared with alternative seed). These scores thus ranged between 0 and 1, with 0 indicating a tower with no features in common with the seed tower type, and 1 indicating a tower with no features in common with the alternative seed type, and 0.5 indicating an equal number of features from the seed and alternative tower types. The average proportional match to the seed was calculated for each chain, again for both the early (1 and 2) and late (3, 4 and 5) generation participants (see Fig. 2). Since there was no effect of tower type in the previous analysis involving matching to seeds, these data were combined for the purpose of this analysis. Using a one-sample t-test against a chance level proportion of 0.5, the unpredictable payoff condition showed significant matching to seed for both early (*t* = 3.535, *df* = 7, *p = *0.010), and late (*t* = 2.961, *df* = 7, *p* = 0.021) generations. In contrast, in the predictable payoff condition, although the trend was in the direction of matching for the early generations, this was not significant (*t* = 1.241, *df* = 9, *p* = 0.246), and there was not even a trend in the direction of matching for the late generations (*t* = −0.331, *df* = 9, *p* = 0.748).

### Height of Cubic and Tripod Condition Towers

**Figure 3:**
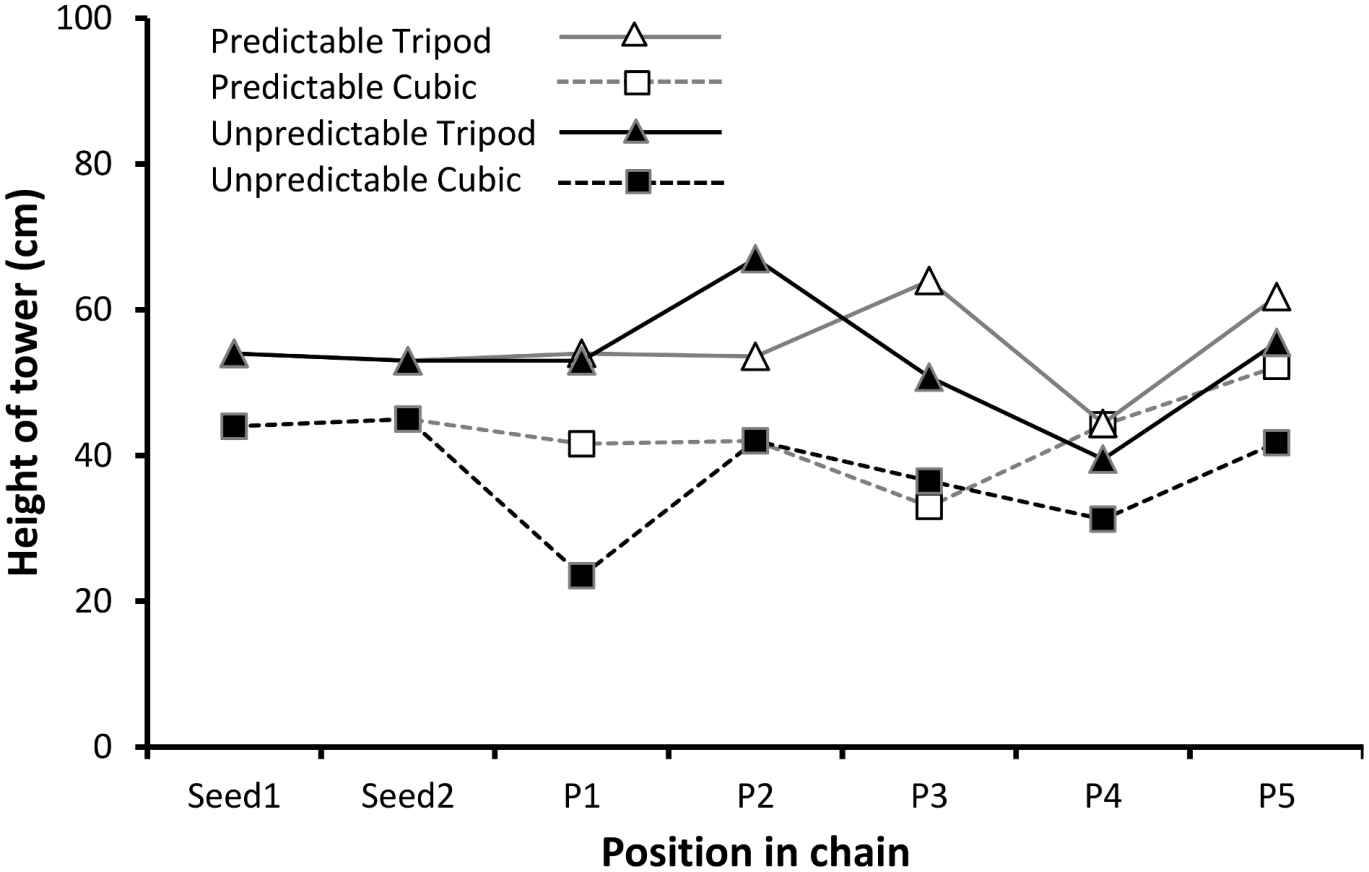
Mean heights of towers built by participants in the four conditions.

Height data were also treated as repeated measures data within chains. Figure 3 displays the mean height data for the four different conditions, for all positions in the chain (including seed towers). As before a 2×2×2 ANOVA was performed, with generation (early; late) as a repeated measures variable, and payoff condition (predictable; unpredictable) and seed tower type (cubic; tripod) as between-subjects variables. Descriptive statistics are displayed in Table 3. There was a main effect of tower type (*F*_1,14_ = 13.158, *p* = 0.003), with towers built in the tripod-seed condition taller than those built in the cubic-seed condition. There was no main effect of either generation (*F*_1,14_ = 0.038, *p = *0.848), or payoff condition (*F*_1,14_ = 0.970, *p = *0.341). Interactions were all non-significant. For generation by payoff condition, *F*_1,14_ = 0.471, *p* = 0.504; for generation by tower type, *F*_1,14_ = 0.609, *p* = 0.448; for payoff condition by tower type, *F*_1,14_ = 0.589, *p* = 0.456; and for the three-way interaction between generation, payoff condition, and tower type, *F*_1,14_ = 0.930, *p* = 0.351. Since the only significant influence on height was seed tower type, data were combined in further analyses of the differences in height between cubic and tripod condition towers.

The seed towers in the tripod conditions were themselves taller than those in the cubic condition (tripod seeds = 54 cm & 53 cm; cubic seeds = 44 cm & 45 cm). However this in itself did not appear to fully account for the difference. Participants in the tripod condition built towers that were comparable in height to the seeds (mean height = 54.49 cm, one-sample t-test against seed height of 53.50 cm: *t* = 0.302, *df* = 44, *p = *0.764), but participants in the cubic condition built towers that were somewhat lower than their original seeds, although this did not quite reach significance (mean height = 39.22 cm, one-sample t-test against seed height of 44.50 cm: *t* = 1.818, *df* = 44, *p = *0.076).

In the cubic condition there was a significant negative correlation between the match-to-seed proportional measure and height (Spearman’s Rho: *r* = −436, *N* = 45, *p = *0.003), suggesting that a tendency to adhere to the cubic design was actually counterproductive. No such relationship was apparent for the tripod condition (Spearman’s Rho: *r* = −.009, *N* = 45, *p = *0.954).

## Discussion

As predicted, greater copying was observed in the unpredictable payoffs condition, compared with the predictable payoff condition, indicating that this manipulation triggered a greater reliance on social learning. This high level of matching meant that in this condition the influence of the particular seed design could be detected in the towers built by even the later participants in that chain, who had not actually observed the seeds for themselves.

The effect of generation on the degree of matching to seeds, common across both payoff conditions, suggests that this effect would become diluted back to chance level within another few generations. Within the context of our experiment therefore, these cultural founder effects are probably fairly transient. However, the unpredictability of the personal payoffs within our experiment was merely relative; the task itself remains a highly transparent one when compared with typical real-world examples of social learning in humans. Consequently, the fact that we can nonetheless identify such a striking difference between the two conditions in our experiment implies that similar real-world effects are in fact liable to be extremely powerful.

Consistent with previous work [14] we also found evidence that the cubic design was less effective than the tripod design. Nonetheless these two designs were equally well copied. Indeed, contrary to our prediction, there was not even any evidence to suggest that the cubic design was eroded more rapidly than the tripod design in the predictable payoffs condition, as the interaction effects involving tower type and payoff condition were non-significant. Interestingly, this implies that participants in the unpredictable payoffs condition were not disadvantaged by their greater reliance on social learning, as the slower rates of change permitted some preservation of both designs, including the more effective tripod approach, whereas both were equally eroded in the predictable payoff chains.

In line with this interpretation, the height data indicates that there was no overall difference between the predictable and unpredictable payoff conditions in terms of this goal measure. Perhaps more surprisingly, there was also no significant interaction between payoff condition and seed tower condition. However, given the small number of replicates involved in this analysis (4 chains cubic predictable, 4 chains tripod predictable, 5 chains cubic unpredictable, 5 chains tripod unpredictable) it is possible that this was attributable to low statistical power. It is worth noting that the trends are in the direction one would expect (Table 3), with the cubic unpredictable towers the least successful in terms of the height goal.

It is likely that the participants in our experiment (in both conditions) were behaving in a highly rational manner, weighing up the likely benefits, and potential risks, associated with copying a previous solution, or attempting something different. In both conditions the payoff was probably relatively predictable for an attempt to reproduce a previous solution, whereas the payoff for a novel solution was less predictable (albeit potentially more profitable). In the unpredictable condition, the uncertainty of the payoff for a novel solution was enhanced by the knowledge that even the height of one’s completed tower was not necessarily a good indication of final payoff. The decision to play safe in this condition, and attempt something similar to a previous solution, is therefore very understandable. Our study illustrates that such reasoning at the individual-level can have population-level consequences in terms of the rates of change in the cultural evolutionary process.

The broader implications of this result may have consequences for our understanding of the extraordinarily powerful influence of social learning in humans, compared with other species. Much of human technology (and indeed that of our hominid ancestors from around 1.5 million years ago, e.g. [15]) would fall within our classification of “unpredictable payoffs”, since tool manufacture is generally separated from use in both time and space. Indeed, Gergely and Csibra [16], [17] have argued that human-unique social learning mechanisms may have evolved in response to the need for preserving “recursive technologies” (such as tools manufactured to produce other tools), where ultimate goals are not immediately obvious to the naïve learner.

Nonetheless, for modern humans (both historically, and in contemporary society) particular decisions can still be classified as having either relatively predictable or relatively unpredictable outcomes for the user, and we might expect to see accordingly fast or slow rates of change in cultural practices. As a simple example, choice of fertilizer, or method of planting, might affect one’s crop yield later in the year, but this connection would likely be relatively opaque to the user, and there would be a significant delay between implementation of a novel technique or product, and feedback on its effectiveness. In contrast, new harvesting machinery might well have clear benefits to the user in terms of time efficiency and/or reduced wastage, and furthermore this would be apparent from first usage. We would hence expect relatively more predictable payoff decisions such as this to exhibit steeper adoption curves for beneficial innovations.

In conclusion, our study indicates that adaptive social learning strategies [8], [9] can result in different rates of cultural change as a consequence of the degree of reliance on social learning versus innovation. Uncertainty about payoffs in particular may be an important predictor of the cultural turnover rate for a given behavior.
